# Application of chitosan/graphene and chitosan/graphene oxide composites for removal of Cu and Pb

**DOI:** 10.1038/s41598-025-13307-6

**Published:** 2025-08-07

**Authors:** Abdel Salam El-Sheikh, Nabil S. Abdelaziz, Khaled S. Amin, Hanan Elhaes, Medhat A. Ibrahim

**Affiliations:** 1 Regional Center for Food and Feed RCFF, Giza, Egypt; 2 Physics Department, Faculty of Science, Mansoura University, Mansoura, 35516 Egypt; 3 Physics Department, Faculty of Science, Al-Azhar University, Cairo, Egypt; 4 Physics Department, Faculty of Women for Arts, Science and Education, Ain Shams University, Cairo, 11757 Egypt; 5 Spectroscopy Department, National Research Centre, 33 El-Bohouth St., Dokki, 12622 Giza Egypt; 6 Molecular Modeling and Spectroscopy Laboratory, Centre of Excellence for Advanced Science, National Research Centre, 33 El-Bohouth St., Dokki, 12622 Giza Egypt

**Keywords:** DFT: B3LYP/LANL2DZ, Chitosan, Graphene, Graphene oxide, And FTIR, Materials science, Physics

## Abstract

Water pollution caused by heavy metals such as lead (Pb) and copper (Cu) represent a critical global challenge due to their toxicity and adverse impacts on both human health and the environment. Among several remediation methods, adsorption using polymer-based sorbents like chitosan (Cs) has emerged as a promising approach. In this study, chitosan interacted with graphene (Gr) and graphene oxide (GrO) to enhance its possible interaction with di-hydrated Pb and Cu. The electronic properties of Cs/Gr and Cs/GrO composites were studied using density functional theory (DFT) at the B3LYP/LANL2DZ level of theory. Physical parameters, including total dipole moment (TDM), HOMO-LUMO energy gap (∆E), and global reactivity descriptors, were calculated. Additionally, molecular electrostatic potential (MESP), density of states (DOS), and frontier molecular orbitals (FMO) were analyzed. The results demonstrated significant improvements in electronic properties, with increased total dipole moment (TDM) values (7.300 Debye for Cs/Gr and 6.311 Debye for Cs/GrO) and reduced ∆E (3.671 eV for Cs/Gr and 2.701 eV for Cs/GrO), indicating enhanced reactivity. Adsorption energies (E_a_) for interactions with di-hydrated Pb and Cu were also evaluated, showing proper binding where E_a_ values of – 13.869 eV for Cs/Gr/di-hydrated Pb, – 13.689 eV for Cs/Gr/di-hydrated Cu, – 12.975 eV for Cs/GrO/di-hydrated Pb, and − 14.211 eV for Cs/GrO/di-hydrated Cu. Quantum Theory of Atoms in Molecules (QTAIM) analysis confirmed E_a_ findings. Cs/GrO composites were synthesized and FTIR spectra were measured and compared to computed vibrational frequencies. This study combines DFT and QTAIM to provide a comprehensive understanding of the selective adsorption behavior of Cs/Gr and Cs/GrO composites for di-hydrated Pb and Cu, supported by FTIR validation of the computational models.

## Introduction

Discharge of heavy metals into the aquatic environment is among the main environmental challenges worldwide. Discharge of heavy metals include copper (Cu) and lead (Pb), into natural water streams. Lead is considered a highly toxic element with significant negative impacts on both the environment and human health^1^. The main sources of lead contamination include the battery industry, paints, electronic products, and the use of lead pipes and taps^2,3^. Copper (II) is considered one of the most common heavy metals in the environment. Fertilizers and pesticides are the main sources of copper in the environment as well as, chemical, pharmaceutical, and paper industries^4^. Copper has severe negative impacts on both human health and ecosystems^5,6^. Adsorption is a widely used method for water pollution treatment due to the availability of various sorbent materials, its simple operation, and the absence of secondary pollution^7,8^. There are many kinds of traditional sorbent materials such as activated carbon and zeolite but their applications are limited^9,10^. Adsorption process depends on the type of sorbents materials therefore, development of low cost and effective adsorbents for heavy metal remediation has a great attention. Hence, polymers as sorbents materials have received much attention due to their advantages, therefore, polymeric sorbents, such as graphene oxide, polyesters and carbon-based materials adsorbents have been used, as well as, natural polymers have received significant attention such as polysaccharides as highly efficient sorbents^11–14^. Polysaccharides derived sorbents including lignin, dextrin, chitosan, and cellulose, are considered an alternative to the traditional sorbents^15^. Graphene (Gr) as a form of carbon adsorbents; it has a single layer of carbon atoms arranged in a hexagonal honeycomb lattice with large surface area, high intrinsic mobility (charge carrier mobility), excellent thermal and electrical conductivity exceeding even that of copper^16^. Graphene possesses remarkable properties that make it a groundbreaking material in the field of materials science. Its exceptional strength, high electrical and thermal conductivity, large surface area, and flexibility have enabled a wide range of advanced applications, particularly in energy storage—such as supercapacitors and advanced batteries—as well as in electronics, including transistors and touchscreen devices^17,18^. Graphene oxide (GO) is a single atomic layer derived from graphite, characterized by the presence of oxygen-containing functional groups such as hydroxyl (-OH) and epoxy groups attached to its carbon structure, with some carboxylic groups located at the edges. These functional groups enhance the hydrophilicity and reactivity of GO, allowing it to interact with various inorganic and organic species through π-π stacking interactions and hydrogen bonding. This versatility makes graphene oxide highly suitable for applications in water purification, sensors, and composite materials^19^. Graphene oxide has gained significant attention in water treatment due to its high surface area, which facilitates chemical interactions with various pollutants, Additionally, it is abundant and low-cost, making it an attractive material for large-scale applications^20,21^. Promising results have been reported in the remediation of water contaminated with emerging pollutants, highlighting graphene oxide’s potential as an efficient adsorbent and membrane material for water purification^22^. The polar functional groups and high surface area of graphene oxide make it highly soluble in water and various organic solvents. In contrast, graphene has low solubility in aqueous solutions due to its nonpolar, hydrophobic nature. This difference in solubility significantly influences their respective applications, especially in fields like water treatment and composite material fabrication^23^. The high negative charge density of graphene oxide in aqueous solutions provides effective adsorption sites for cationic pollutants, such as methylene blue dye. This is due to the presence of oxygen-containing functional groups (e.g., carboxyl, epoxy, and hydroxyl groups) on the graphene oxide surface, which provides a strong negative charge that facilitates electrostatic attraction and binding of positively charged species^24^. Graphene oxide possesses physicochemical properties such as mechanical strength and greater adsorption power^25^. Chitosan is a linear polysaccharide chemical formula (C_6_H_11_NO_4_)_n_ composed of D-glucosamine (deacetylated units) and N-acetyl-D-glucosamine (acetylated units) linked by β-(1_4) glycosidic bonds, and is a positively charged polysaccharide^26^. Chitosan (Cs) is a renewable biopolymer, biocompatible, non-toxic^27–29^. Chitosan is widely produced by partial or complete deacetylation, which involves removing acetyl groups from the N-acetyl-D-glucosamine units of chitin that found in shrimp shells, crabs, prawns, and krill. Chitosan is considered the second most abundant natural polymer on earth after cellulose^30^. Chitosan has functional groups such as hydroxyl (-OH) and amino (-NH₂) therefore it has high active adsorption sites affinity comparing to other poly saccharides, and has been used as coordination sites^31,32^. Under acidic conditions the NH_2_ group inside chitosan is protonated -NH_3_+, so it is considerfed a natural cationic polymer, and thus significantly adsorbs negatively charged contaminants^33^. Nevertheless, chitosan degrades over time and exhibits mechanical weakness, which limits its practical applications^34^. Molecular modeling is a computational technique used to model and simulate the behavior of molecules at atomic level to help understanding molecular structures, interactions and properties using mathematical equations and algorithms. It provides scientists with information about molecular interactions and reactivity that can’t be observed experimentally^35^. Molecular modeling predicts the molecular structures properties (molecular geometry), calculates spectroscopic properties, and predicts electronic properties^36,37^. Theoretical basis of molecular modeling based on methods such as quantum mechanics, classical mechanics and statistics thermodynamics^38^. Density Functional Theory (DFT) is a quantum mechanics method, requiring comparable computational effort, DFT calculations have been used to study the electronic structure of atoms and molecules based on electron density instead of wave function method^39^.

The aim of this study is to apply molecular modeling, at Density Functional Theory (DFT) with B3LYP/LANL2DZ level of theory, to evaluate the electronic properties of modified Cs with Gr and GrO in order to evaluate the adsorption efficiency. Their possible interactions with di-hydrated Pb and di-hydrated Cu metals were investigated using multiple approaches. The calculated parameters included the total dipole moment (TDM), the HOMO-LUMO energy gap, and frontier molecular orbitals. Moreover, molecular electrostatic potential (MESP) maps, density of states (DOS), and global reactivity descriptors were also calculated. Adsorption energy (Ea) was calculated for these models after heavy metal adsorption. Quantum Theory of Atoms in Molecules (QTAIM) analysis was conducted to examine the nature of the bonds formed with Pb and Cu and to assess the stability of the structures. For experimental verification Cs and Cs/GrO samples were prepared, and Fourier-transform infrared (FTIR) measurements were performed to compare both the experimental and the computed IR. The integration of DFT, QTAIM, and FTIR validation offers new insights into their selective adsorption behavior, an aspect not extensively addressed in previous works on removal of heavy metals using Cs/GrO composites.

## Calculation details

To investigate the interaction of the proposed structures with the heavy metals Cu, Pb using Gaussian 09 software package from Gaussian, Inc., Wallingford, CT, USA^40^, for structure optimization and molecular characteristic calculations at the Molecular Spectroscopy and Modeling Unit, Centre of Excellence for Advanced Science, National Research Centre, Cairo, Egypt. Optimization and calculations were carried out using DFT: B3LYP methods at LANL2DZ basis set^41–43^. This combination was chosen for its balance between computational efficiency and accuracy, especially for systems involving transition metals. LANL2DZ includes effective core potentials (ECPs), which incorporate relativistic corrections necessary for heavier elements such as Pb and Cu^44,45^. Physical parameters were calculated including TDM, HOMO/LUMO band gap energy (∆E). MESP contours were mapped as well as the vibrational frequencies. The density of states DOS was calculated for all the studied structures at same level of theory. Global reactivity descriptors including ionization potential (I), electronic affinity (A), chemical potential (µ), chemical hardness (η), absolute chemical softness (S) and electrophilicity index (ω) were calculated using the following Eqs.^46,47^:

$$\begin{gathered} {\text{I }}={\text{ }} - {{\text{E}}_{{\text{HOMO}}}} \hfill \\ {\text{A}}\,=\,{{\text{E}}_{{\text{LUMO}}}} \hfill \\ \mu ={\text{ }} - \left( {{\text{I}}\,+\,{\text{A}}} \right)/{\text{2}} \hfill \\ \eta =\left( {{\text{I}} - {\text{A}}} \right)/{\text{2}} \hfill \\ {\text{S}}\,=\,{\text{1}}/\eta \hfill \\ \omega \,=\,{\mu ^{\text{2}}}/{\text{2}}\eta \hfill \\ \end{gathered}$$

QTAIM were carried out using Multiwfn and VMD software^48,49^. Adsorption energy (E_a_) was calculated for the studied structures using following equation^50^:

$${{\text{E}}_{\text{a}}}={\text{ }} - {\text{ }}\left[ {{{\text{E}}_{{\text{system}}}} - {\text{ }}\left( {{{\text{E}}_{{\text{adsorbent}}}}+{\text{ }}{{\text{E}}_{{\text{adsorbate}}}}} \right)} \right]$$

## Materials and methods

### Materials

Chitosan with a deacetylation degree of 90% ±5 was purchased from Chitosan Egypt LLC. Sulfuric acid (96%) was supplied by Scharlau, while hydrogen peroxide (30%) was obtained from PIOCHEM. Sodium hydroxide and ethanol were purchased from El Nasr Pharmaceutical Chemicals Co., Cairo, Egypt. Citric acid (99.5%), sodium hydroxide (≥ 97%), phosphoric acid (85%), potassium permanganate (99%), and graphite powder were provided by Fisher Chemical. Distilled water and deionized water (DI) Milli-Qwater system were used for sample preparation.

### Samples preparation

#### Synthesis of graphene oxide

Graphene oxide (GrO) was synthesized using the modified Hummer’s method. Three grams of graphite flakes were mixed with 360 ml of sulfuric acid and 40 ml of phosphoric acid in a 9:1 ratio, then the mixture was stirred continuously. The mixture then cooled in an ice bath, then 18 g of KMnO₄ was added gradually. The mixture’s color shifted from black (graphite) to dark olive green, while the temperature was kept between 0 and 5 °C in order to reduce exothermic reactions. Afterwards, 400 ml iced deionized water with a 30% concentration of hydrogen peroxide (H₂O₂) was mixed together after the mixture had been stirred overnight at 40 °C. This resulted in a color change from violet to light brown. The supernatant was discarded after the mixture was filtered via centrifugation at 10,000 rpm. The filtrate obtained was subjected to drying in an oven at a temperature of 70 °C for five hours.

#### Synthesis of Cs/GrO composite

Chitosan (Cs) and graphene oxide (GrO) were mixed to form a composite using the casting method^51,52^. In particular, 50 ml of deionized water containing 2% acetic acid was used to dissolve 0.25 g of Cs while being constantly stirred. Two samples had been prepared, one of them consist of pure Cs and the other consist of 90% Cs and 10% GrO. At 70 °C, both samples were stirred for one hour. The final composite film was created by drop-casting the resultant solutions into petri dishes in air dry for five days at room temperature.

### FTIR spectroscopy

The spectrometer VERTEX 70 (Bruker Corporation, Germany) in conjunction with Platinum Diamond ATR, which uses a diamond disc as an internal reflection element, was used to gather ATR-FTIR spectral data in the 4000 –400 cm^− 1^ range at the National Research Centre (NRC), Cairo, Egypt. The spectra were gathered with a 4 cm^− 1^ spectral resolution. 35 scans were performed on each spectrum. A 4 cm^− 1^ resolution test of the backdrop versus air was conducted using the same parameters.

## Results and discussion

### Building and optimization of model molecules

**Fig. 1:**
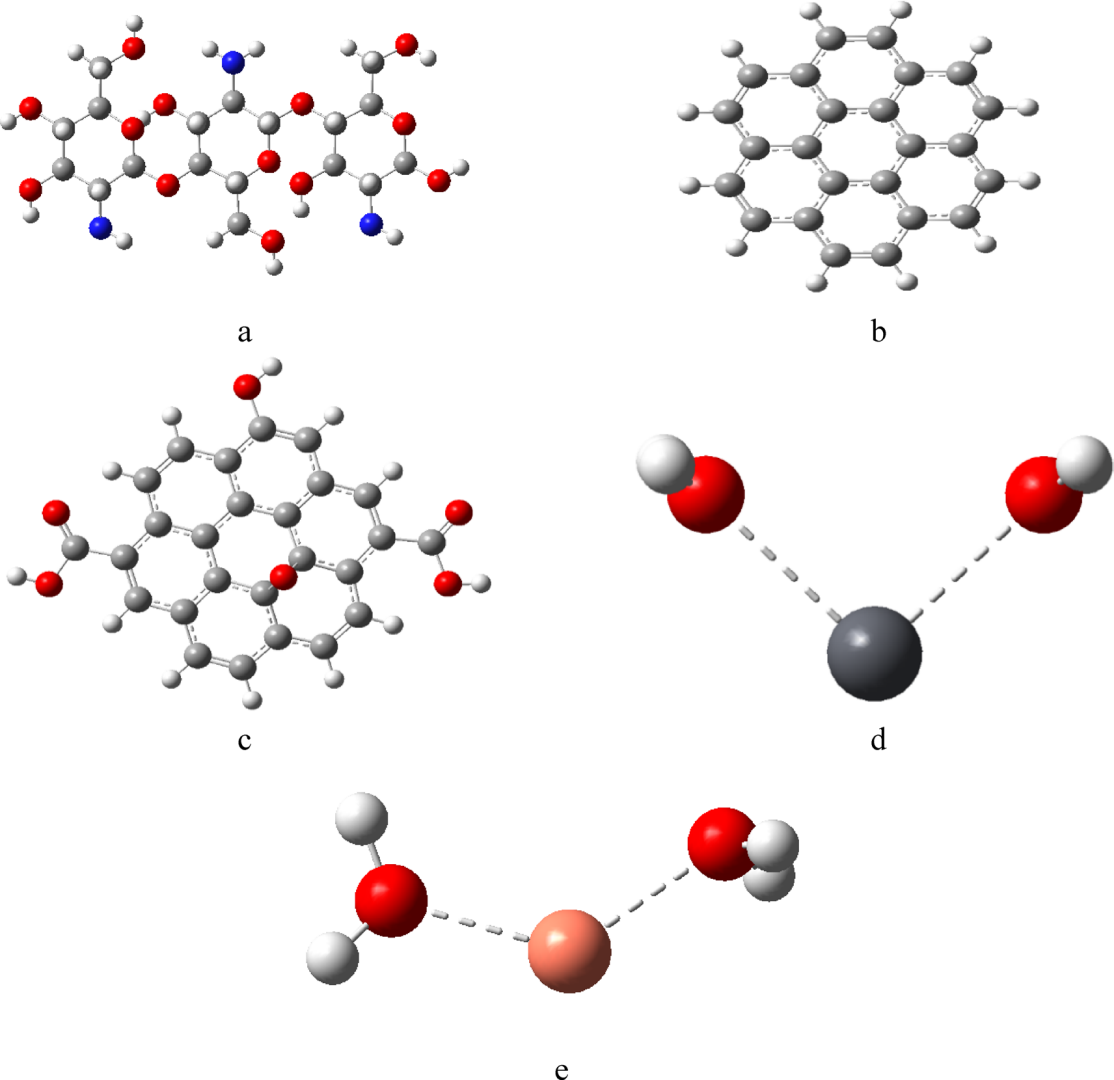
Optimized model molecules for the studied structures whereas; (**a**) Cs, (**b**) Gr, (**c**) GrO, (**d**) di-hydrated Pb and (**e**) di-hydrated Cu.

**Fig. 2:**
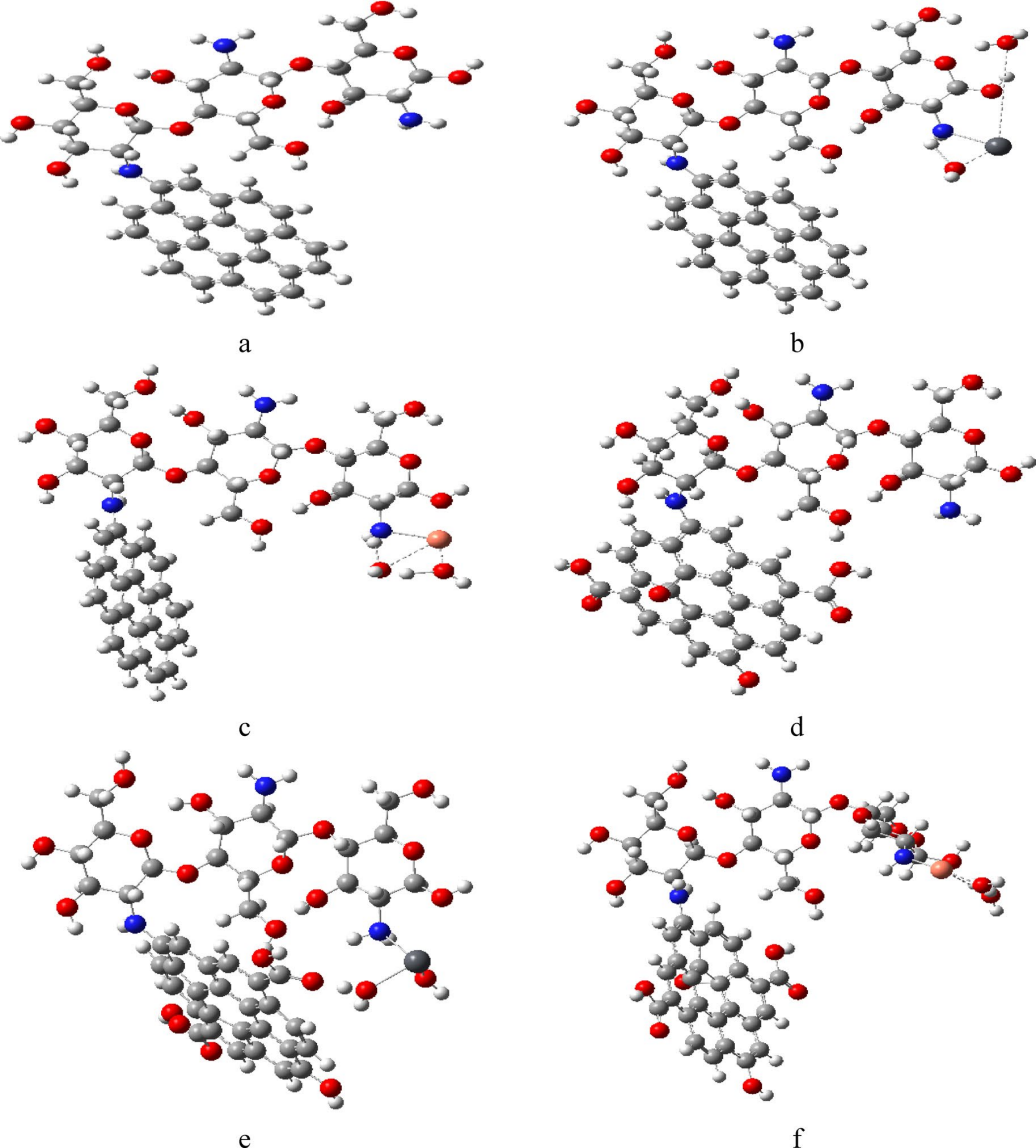
Optimized model molecules for the studied structures whereas; (**a**) Cs/Gr, (**b**) Cs/Gr/di-hydrated Pb, (**c**) Cs/Gr/di-hydrated Cu, (**d**) Cs/GrO, (**e**) Cs/GrO/di-hydrated Pb and (**f**) Cs/GrO/di-hydrated Cu.

The proposed models of the studied molecules structures were conducted, as shown in Fig. 1 where 1a is chitosan (Cs) which consists of three units, contains hydroxyl -OH and amino -NH_2_ function groups, Fig. 1b represents graphene (Gr) it has a single layer of carbon atoms, Fig. 1c represents graphene oxide (GrO) which contains hydroxyl (-OH), epoxy group -O- on the basal plane and carboxyl COOH at the edges of (GrO) attached to the carbon structure. Heavy metals in aquatic system Pb and Cu were modeled in Fig. 1d as di-hydrated Pb, and Fig. 1e as di-hydrated Cu. To investigate the adsorption affinity of chitosan as sorbent material, it was combined with graphene and graphene oxide forming a chitosan graphene (Cs/Gr) and chitosan graphene oxide composite (Cs/GrO). Cs interacts with Gr and GrO through N atom of NH_2_ group. Optimization of all proposed molecules was carried out using DFT method, B3LYP and LANL2DZ basis set at the ground state energy to get the stable geometric configuration for the designed molecules. For the Cs/Gr composite, the N atom of the amino group of Cs interacted with Gr as shown in Fig. 2a, furthermore Cs/Gr composite interacted with di-hydrated Pb, and Cu weakly with N atom of the amino group of Cs as shown in Fig. 2b, c respectively. Similarly, Cs and GrO interaction occur via the amino group forming a complex as shown in Fig. 2d. Finally, the optimized models shown in Fig. 2e and f display the interaction of Cs/GrO with di-hydrated Pb and di-hydrated Cu respectively.

### Total dipole moment (TDM) and band gap energy (∆E)

The reactivity of the studied molecules as adsorbents can be evaluated using two key parameters, TDM and ΔE, as summarized in Table 1. TDM quantifies charge separation within a molecule or system. A higher TDM combined with a lower ΔE generally correlates with enhanced molecular reactivity^53,54^. The calculated TDM values ranged from 0.000 to 7.300 Debye. Composites such as Cs/Gr and Cs/GrO exhibited higher TDM values compared to their individual components (Cs, Gr and GrO). Interaction with metals further increased TDM, particularly with Pb composites like Cs/Gr/di-hydrated Pb, Cs/Gr/di-hydrated Cu, Cs/GrO/di-hydrated Pb, and Cs/GrO/di-hydrated Cu showed significant improvements. Band gap analysis revealed a systematic reduction in ΔE across composites. Pristine materials showed ΔE values of 6.908 eV (Cs), 4.025 eV (Gr), and 2.906 eV (GrO). Composite formation significantly lowered ΔE to 3.671 eV (Cs/Gr) and 2.701 eV (Cs/GrO). Metal interaction further reduced ΔE, with Pb-containing composites exhibiting the most dramatic decreases: 0.675 eV (Cs/Gr/Pb) and 0.919 eV (Cs/GrO/Pb). In contrast, Cu-modified systems showed moderate reductions (2.613 eV for Cs/Gr/Cu, 1.937 eV for Cs/GrO/Cu).

**Table 1:** B3LYB: LANL2DZ calculated TDM as Debye and energy gap ∆E as eV for the studied structures.

Structure	∆E (eV)	TDM (Debye)
Cs	6.908	5.884
Gr	4.025	0.000
GrO	2.906	2.890
Cs/Gr	3.671	7.300
Cs/Gr/ di-hydrated Pb	0.675	9.220
Cs/Gr/ di-hydrated Cu	2.613	8.111
Cs/GrO	2.701	6.311
Cs/GrO/di-hydrated Pb	0.919	13.309
Cs/GrO/di-hydrated Cu	1.937	3.629

### Frontier molecular orbitals (FMOs) and density of States (DOS)

**Fig. 3:**
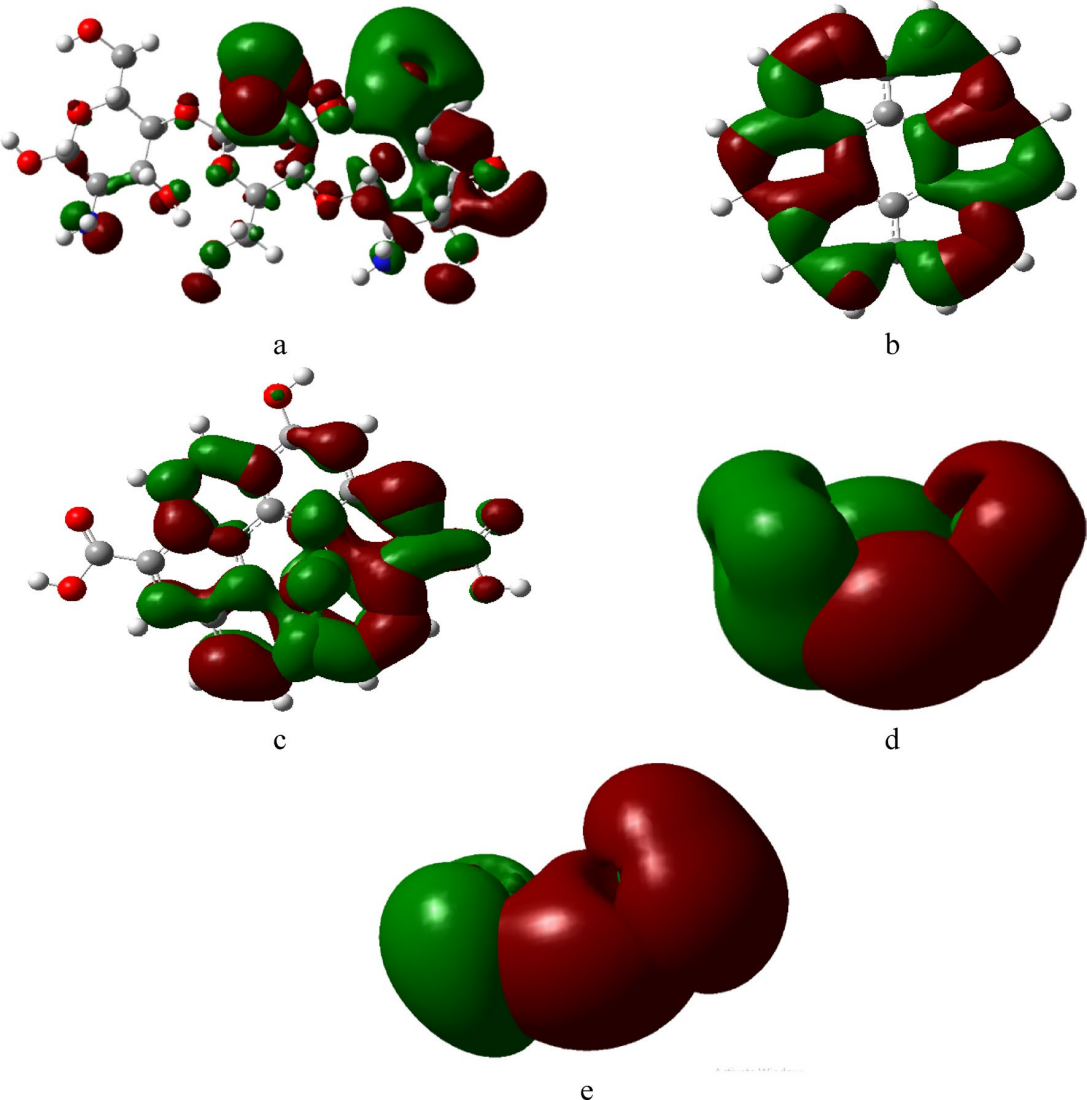
B3LYP/LANL2DZ Calculated HOMO/LUMO frontier molecular orbitals for studied structures whereas; (**a**) Cs, (**b**) Gr, (**c**) GrO, (**d**) di-hydrated Pb and (**e**) di-hydrated Cu. This figure is generated with GaussView 5.0 software implemented in G09 softcode^40^.

**Fig. 4:**
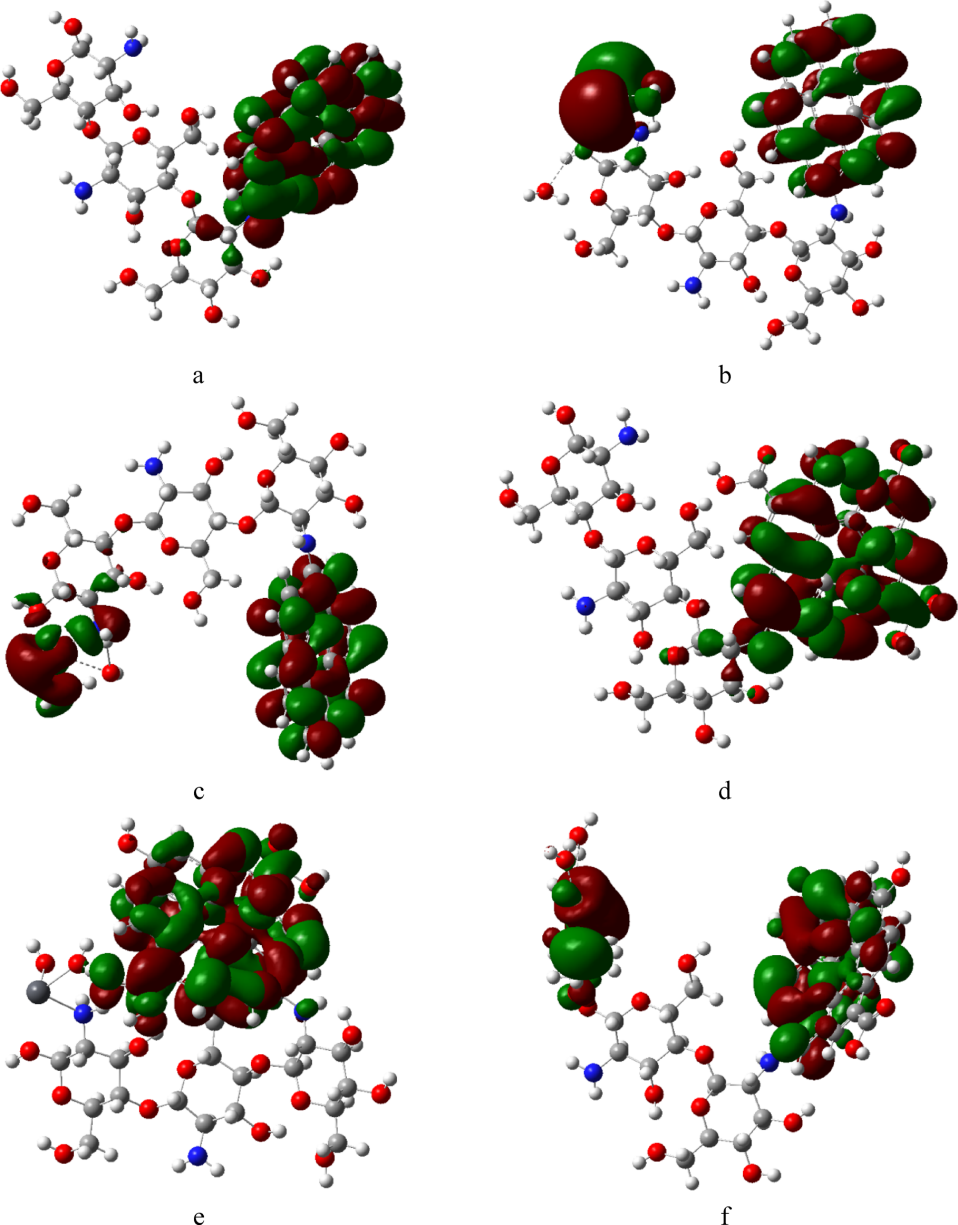
B3LYP/LANL2DZ Calculated HOMO/LUMO frontier molecular orbitals for studied structures whereas; (**a**) Cs/Gr, (**b**) Cs/Gr/di-hydrated Pb; (**c**) Cs/Gr/di-hydrated Cu, (**d**) Cs/GrO; (**e**) Cs/GrO/di-hydrated Pb and (**f**) Cs/GrO/di-hydrated Cu. This figure is generated with GaussView 5.0 software implemented with G09 softcode^40^.

FMOs refer to the highest occupied molecular orbital (HOMO) and lowest unoccupied molecular orbital (LUMO) of molecules, which help understands the chemical reactivity such as charge transfer and photochemistry. Studying FMOs help for predicting reactivity and intermolecular interactions^55^. HOMO represents the most energetic electrons available for bonding (electron donor), while LUMO represent the lowest energy orbital that accept electrons and their difference (band gap) impacts electronic and optical properties^56^. Studied structures presented in Fig. 3 were calculated at same level of theory, where green orbitals denoted to negative region while red denoted to positive region. HOMO/LUMO in Cs/Gr and Cs/GrO shown in Fig. 4a, d are localized on Gr and GrO surface. After the adsorption of Pb and Cu, redistribution took place for HOMO/LUMO orbitals, whereas for Cs/Gr they localized around the heavy metal atoms, as shown in Fig. 4b, c, while HOMO orbitals for Cs/GrO were localized around GrO and Cu atom as shown in Fig. 4f. In contrast HOMO orbitals for Cs/GrO/di-hydrated Pb were localized around GrO Fig. 4e. These results indicate a better charge transfer mechanism for Cs/Gr with Pb, Cu while Cs/GrO exhibits a better charge transfer for Cu than Pb.

**Fig. 5:**
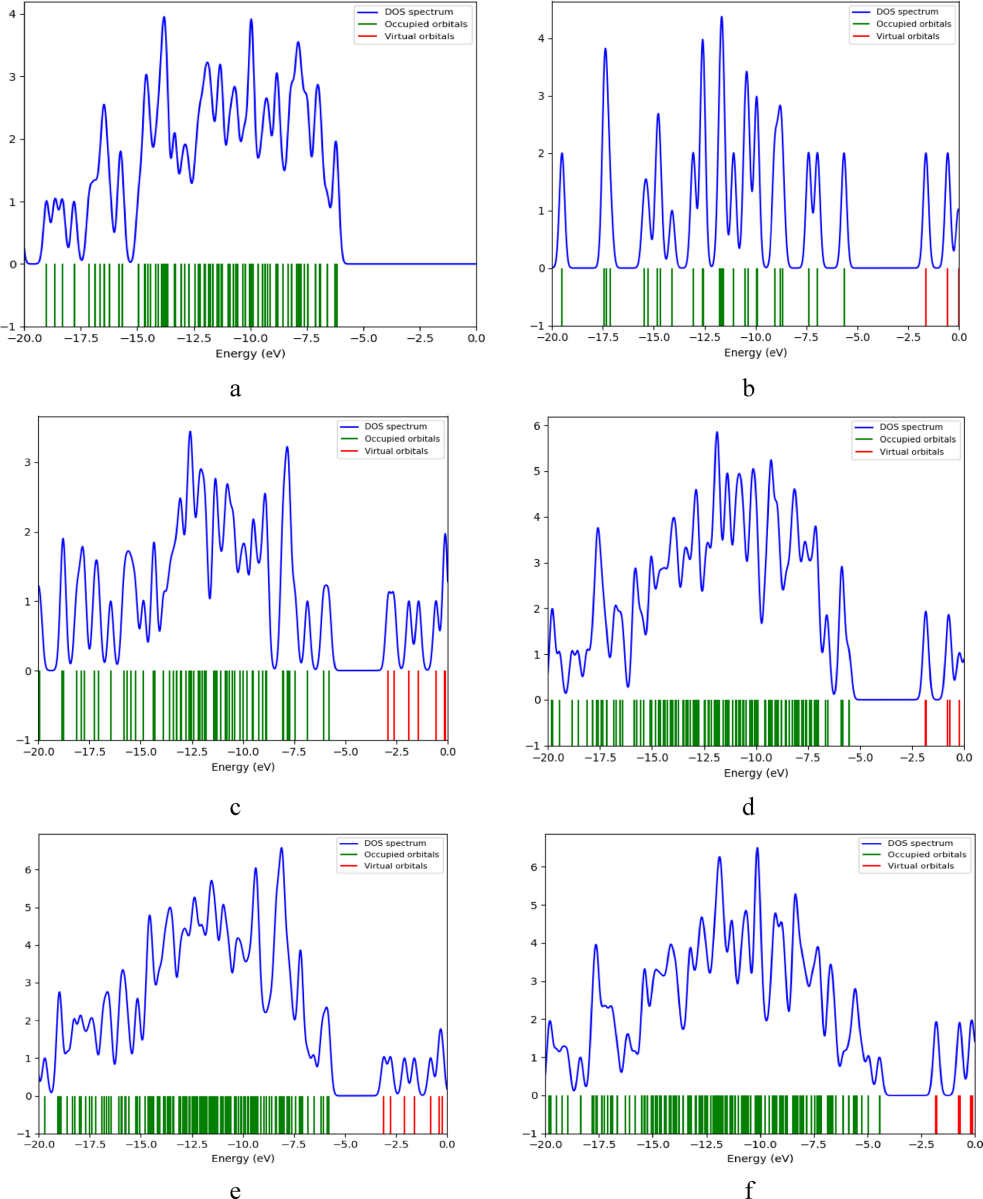

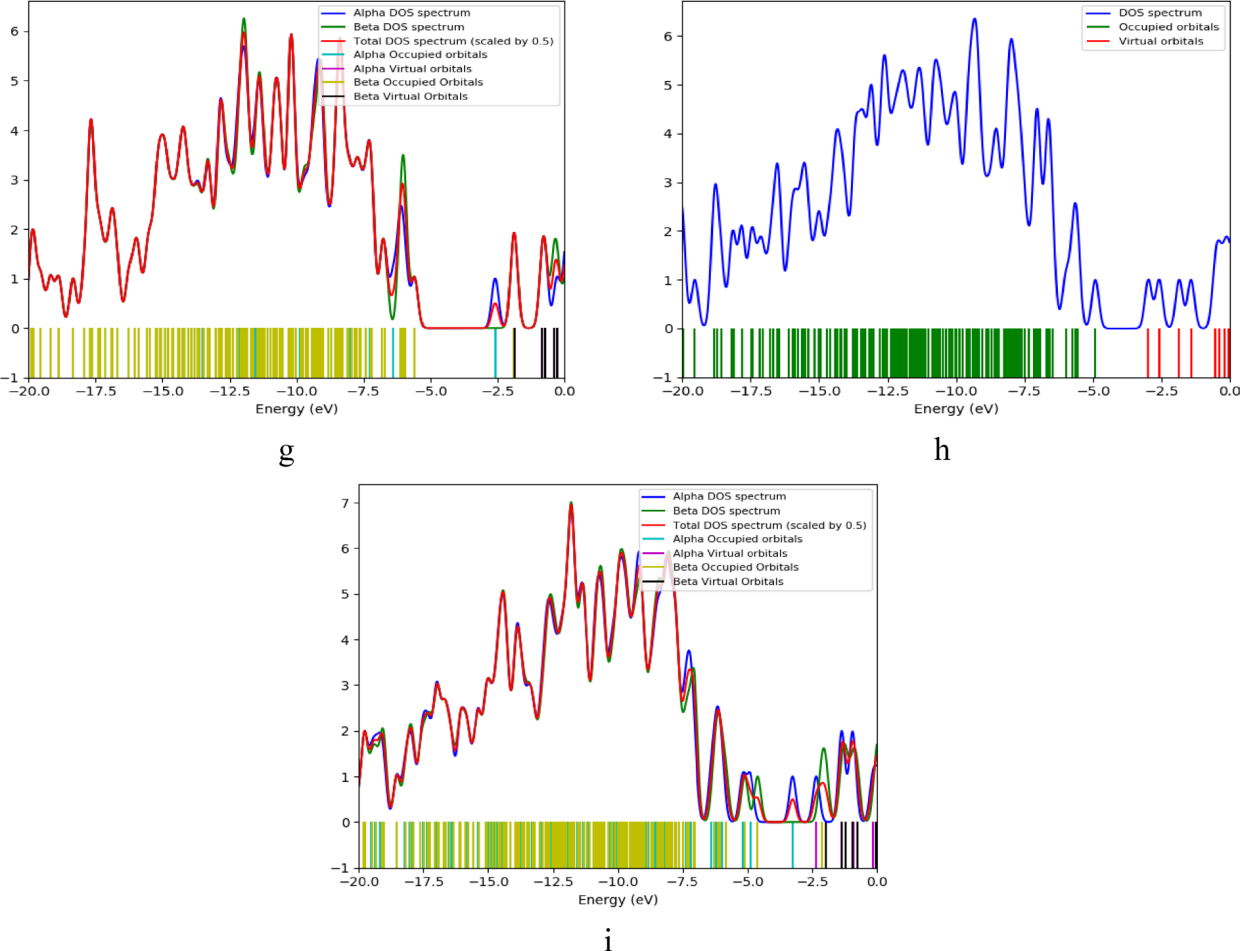
B3LYP/LANL2DZ Calculated DOS for the studied structures whereas; (**a**) Cs, (**b**) Gr, (**c**) GrO, (**d**) Cs/Gr, (**e**) Cs/GrO, (**f**) Cs/Gr/di-hydrated Pb, (**g**) Cs/Gr/di-hydrated Cu, (**h**) Cs/GrO/di-hydrated Pb and (**i**) Cs/GrO/di-hydrated Cu.

DOS can be defined as the number of available electronic states per unit energy per unit volume. It is important to understand electrical, thermal and optical properties of metals, semiconductors and insulators. Figure 5 represents DOS plots for Cs, Gr, GrO, Cs/Gr, Cs/GrO, Cs/Gr/di-hydrated Pb, Cs/Gr/di-hydrated Cu, Cs/GrO/di-hydrated Pb and Cs/GrO/di-hydrated Cu, respectively. In these DOS plots, blue lines represent the density of electronic states as a function of energy, indicating energy level that states are concentrated. Green lines represent the energy levels occupied by electrons in occupied orbitals, which are located below Fermi level, whereas red lines represent unoccupied energy levels or virtual states which are located above Fermi level. Band gap is the energy between the highest occupied molecular orbital (HOMO) and the lowest unoccupied molecular orbital (LUMO). Notably, Pb induces orbital polarization, splitting unoccupied states into distinct aplha (spin-up) and beta (spin-down) orbitals. The most significant reduction in band gap occurs in the Cs/Gr and Cs/GrO composites, which exhibit enhanced electronic properties compared to Cs alone.

### Molecular electrostatic potential (MESP)

**Fig. 6:**
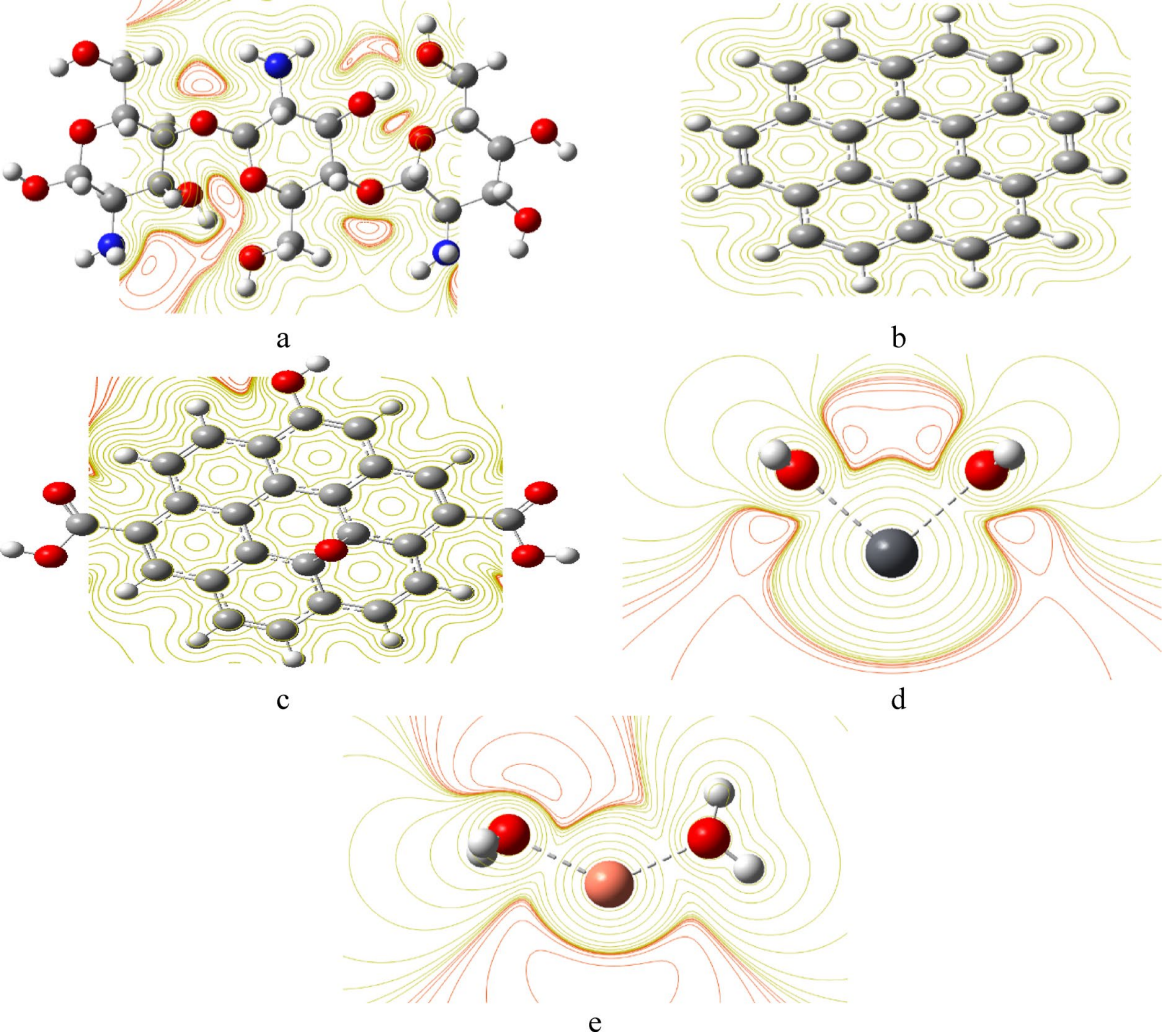
Calculated B3LYP: LANL2DZ MESP for studied structures whereas; (**a**) Cs, (**b**) Gr; (**c**) GrO, (**d**) di-hydrated Pb and (**e**) di-hydrated Cu. This figure is generated with GaussView 5.0 software implemented in G09 softcode^40^.

**Fig. 7:**
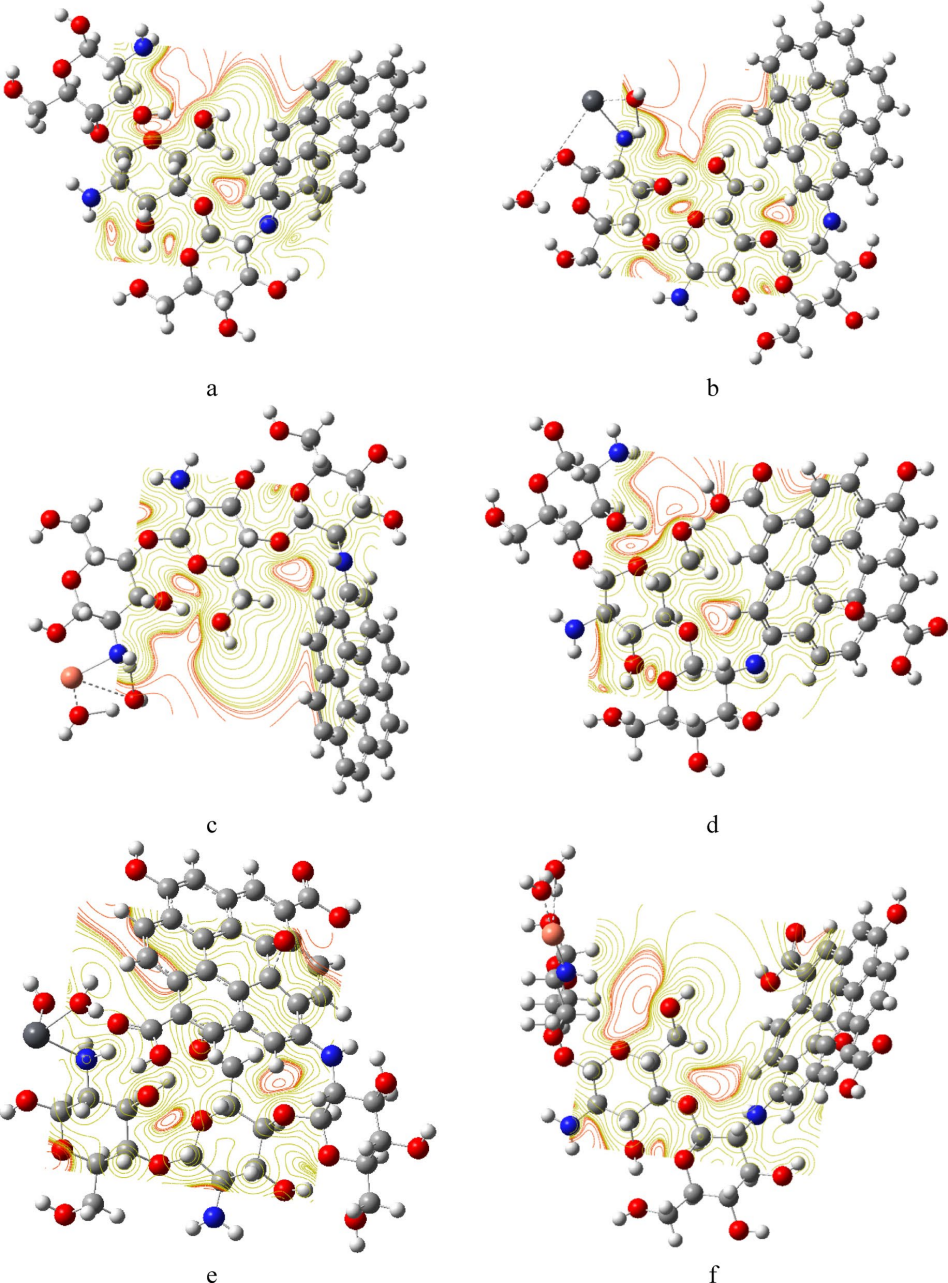
Calculated B3LYP: LANL2DZ MESP for studied structures whereas; (**a**) Cs/Gr, (**b**) Cs/Gr/di-hydrated Pb; (**c**) Cs/Gr/di-hydrated Cu, (**d**) Cs/GrO; (**e**) Cs/GrO/di-hydrated Pb and (**f**) Cs/GrO/di-hydrated Cu. This figure is generated with GaussView 5.0 software implemented in G09 softcode^40^.

MESP represents the electrostatic potential arising from the spatial distribution of electronic and nuclear charges within a molecule. MESP is often used to visualize charge distribution to predict active sites and to understand intermolecular interactions. MESP utilizes a spectrum of color codes to represent the charge distribution, where red areas indicate negative potential and are rich in electron density, such as lone pairs of electronegative atoms. Blue areas indicate positive potential and represent electron-deficient regions, often near hydrogen atoms or electropositive groups, while yellow represents a neutral potential, following the color spectrum: Red > Orange > Yellow > Green > Blue^53^. MESP generated maps are presented in Figs. 6 and 7. Figure 6 illustrates the MESP of Cs, Gr and GrO. The active sites were identified as the red regions around the oxygen atoms for Cs and the surface edge of GrO, as shown in Fig. 6a, c. Gr has a neutral yellow surface, as shown in Fig. 6b. The generated MESP maps for di-hydrated metals are indicated in Fig. 6d and e, both maps indicating the reactivity of hydrated metals.

Similarly, Fig. 7a, d shows that the intensity of the red color in the regions along the oxygen atoms of composites Cs/Gr and Cs/GrO is greater than that of Cs and GrO individually, as shown in Fig. 7b, c. The previous results indicated that, the composites have more active sites for interaction compared to the individual molecules. The generated MESP maps for Cs/GrO/di-hydrated Pb indicated in Fig. 7e and maps for Cs/GrO/di-hydrated Cu in Fig. 7. It is clear that, as far as the composites interact with hydrated metals the active sites increased as shown in Fig. 7e, f.

### Global reactivity descriptors

Global reactivity descriptors calculated at B3LYP/LANL2DZ level as recorded in Table 2, reveal systematic changes in the electronic behavior of Cs, Gr, GrO, individually, and after interaction with heavy metals. Cs shows the highest ionization potential (6.181 eV), while GrO displays the greatest electron affinity (2.901 eV). Formation of composites lowers Cs’s ionization potential to 5.541 eV for Cs/Gr and 5.796 eV for Cs/GrO, suggested enhanced donor ability. Upon interaction with heavy metals a further decrease to I (2.584 eV) in Cs/Gr/di-hydrated Pb and 3.259 eV in Cs/GrO/di-hydrated Pb. The chemical potential (µ) becomes less negativity based on metal binding where Cs/Gr shifts from − 3.705 to −  2.247 eV (Pb) and − 3.134 eV (Cu), and Cs/GrO shifted from − 4.446 to −  2.800 eV (Pb) and − 3.941 eV (Cu). Hardness (η) decreased and softness (S) increases after composite formation indicating greater reactivity, η and S also change after the interaction with the studied heavy metals with di-hydrated Pb system having higher S and lower η. Finally, the electrophilicity index (ω) increases for composites where Cs/Gr reaches 3.740 eV (compared to 0.791 eV for Cs), and 7.318 eV for Cs/GrO. Metal adsorption further raised ω. These trends correlate well with ΔE reduction and MESP.

**Table 2:** B3LYB/LANL2DZ calculated global reactivity descriptors as eV for the studied structures.

Structure	I (eV)	A (eV)	µ (eV)	η (eV)	S (eV^− 1^)	ω (eV)
Cs	6.181	– 1.312	– 2.434	3.746	0.267	0.791
Gr	5.652	1.627	– 3.640	2.013	0.497	3.291
GrO	5.807	2.901	– 4.354	1.453	0.688	6.524
Cs/Gr	5.541	1.870	– 3.705	1.836	0.545	3.740
Cs/GrO	5.796	3.095	– 4.446	1.351	0.740	7.318
Cs/Gr/di-hydrated Pb	2.584	1.909	– 2.247	0.337	2.964	7.479
Cs/Gr/di-hydrated Cu	4.440	1.828	– 3.134	1.306	0.766	3.759
Cs/GrO/di-hydrated Pb	3.259	2.340	– 2.800	0.459	2.177	8.531
Cs/GrO/di-hydrated Cu	4.909	2.9718	– 3.941	0.969	1.032	8.014

### Adsorption energy (E_a_)

The adsorption energy (E_a_) provides the order of magnitude of interaction between an adsorbent (the surface) and an adsorbate (the molecule on the surface). E_a_ provides the variation in energy in the process of adsorption, where a more negative value indicates a stronger interaction and a more stable adsorbed state. The calculation of E_a_ is also crucial for determining the selectivity of the composite materials towards different interacting metal species. The calculated E_a_​values are listed in Table 3 in eV. The negative values of E_a_ indicate an exothermic adsorption process, where energy is released upon the adsorbate binding to the surface. E_a_ values suggest that Cs/GrO composite exhibits greater and more favorable adsorption interaction with di-hydrated Cu (−  14.211 eV) compared to Cs/Gr (−  13.689 eV). Conversely, the Cs/Gr composite shows stronger interaction with di-hydrated Pb (−  13.869 eV) than Cs/GrO composite (−  12.975 eV). This implies that Cs/Gr has a notable selectivity for the adsorption of Pb, while Cs/GrO composite may have a stronger affinity for Cu adsorption. The strong adsorption capabilities observed are consistent with experimental findings on Cs/GrO composites for heavy metal removal, including Pb and Cu^57,58^.

**Table 3:** B3LYB/LANL2DZ calculated E_a_ as eV for the studied structures.

Structures	E_a_ (eV)
Cs/Gr/di-hydrated Pb	– 13.869
Cs/Gr/di-hydrated Cu	– 13.689
Cs/GrO/di-hydrated Pb	– 12.975
Cs/GrO/di-hydrated Cu	– 14.211

### Quantum theory of atoms in molecules (QTAIM)

**Fig. 8:**
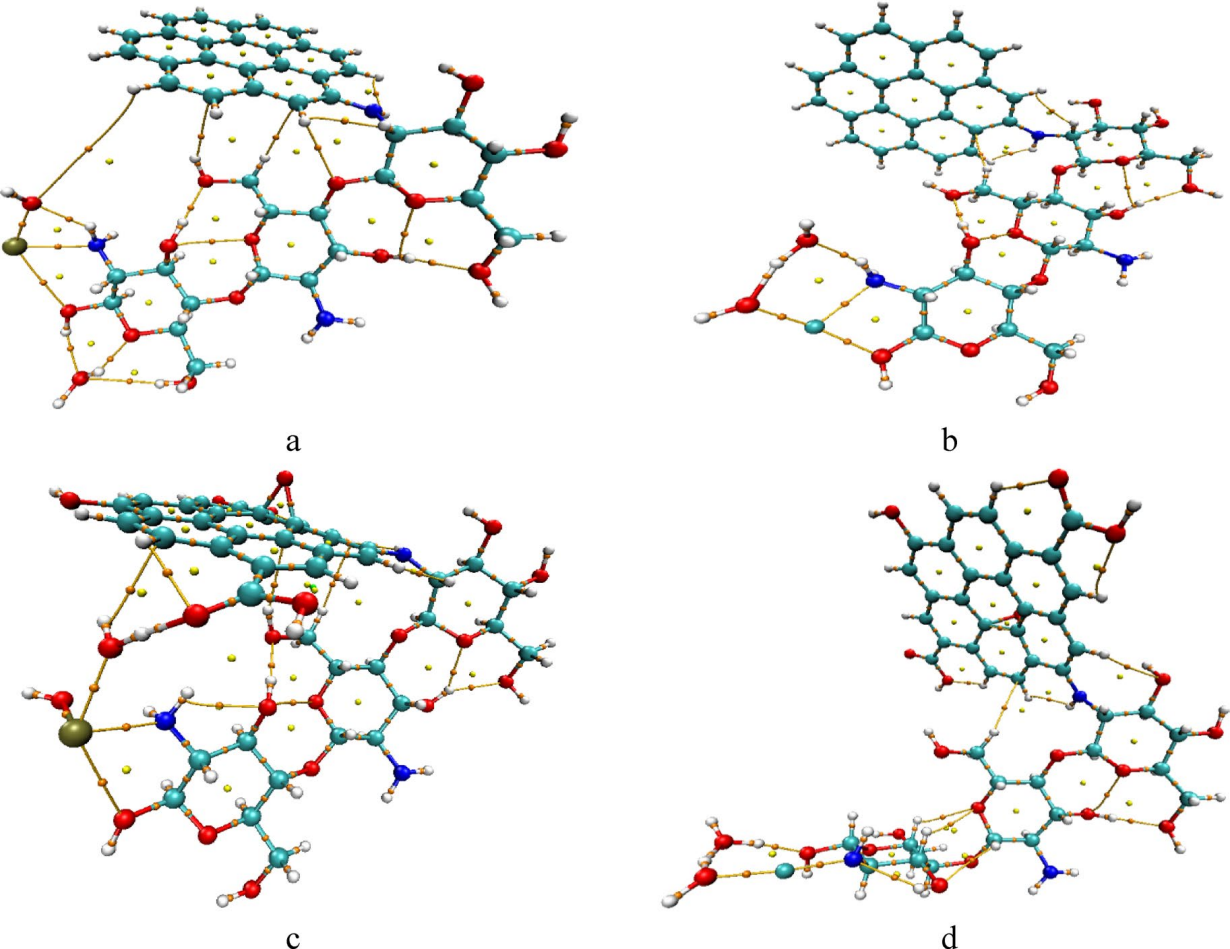
B3LYP/LANL2DZ calculated QTAIM topology for the studied composites whereas; (**a**) Cs/Gr/di-hydrated Pb, (**b**) Cs/Gr/di-hydrated Cu, (**c**) Cs/GrO/di-hydrated Pb and (**d**) Cs/GrO/di-hydrated Cu. This figure is generated using Multiwfn and VMD software.^48,49^.

QTAIM analysis was performed on the composites interacting with di-hydrated Pb and Cu to investigate the nature of their interactions, chemical bonds, and to understand the behavior of the adsorption processes by assessing bonding strength between interacting atoms based on electron density ρ(r) at bond critical points (BCPs)^59,60^. A high electron density (ρ(r) > 0.2 a.u.), coupled with a negative Laplacian (∇^2^ρ(r) < 0) and energy density (H(r) < 0), are indicative of a shared interaction characteristic of strong covalent bonds. Conversely, lower electron density suggests weaker interactions, which can range from polar-covalent bonds (characterized by a more depleted electron density but still some degree of sharing) to closed-shell interactions like van der Waals forces or hydrogen bonds. Figure 8 shows QTAIM topology for the composites interacting with di-hydrated Pb and di-hydrated Cu. Table 4 records the values of ρ(r), ∇²ρ(r), and H(r) for the bonds formed between the N atom of the composite and the heavy metals (N-Pb and N-Cu) in Cs/Gr/di-hydrated Pb, Cs/Gr/di-hydrated Cu and Cs/GrO/di-hydrated Pb, Cs/GrO/di-hydrated Cu. In case of Cs/Gr interacting with di-hydrated Pb and Cu, the values of ρ(r) (0.053 a.u.), the positive ∇²ρ(r) (0.162) and negative H(r) (– 0.837 a.u.) suggest a stronger bond with Pb compared to Cu. In case of Cs/GrO interacting with di-hydrated Pb and di-hydrated Cu, ρ(r) (0.117 a.u.), ∇²ρ(r) of 0.581 a.u. and the negative H(r) values shows stronger bond with Cu. The presence of bond paths, BCBs and the new formed hydrogen bonds as shown in Fig. 8 infer stability for all structures. The electron density (ρ), Laplacian (∇²ρ) and H(r) confirmed the covalent bond between the Cs/Gr and Cs/GrO composites. The QTAIM analysis indicates that Cs/Gr interacted with the heavy metal is stronger with Pb, while Cs/GrO, the interaction with Cu is stronger, which aligns well with the results obtained from E_a_ results.

**Table 4:** B3LYB: LANL2DZ calculated electron density ρ(r), ∇²ρ(r), and energy density H(r) for Cs/Gr and Cs/GrO interacted with di-hydrated Pb and di-hydrated Cu.

Structure	ρ(*r*) (a.u.)	∇²ρ(*r*) (a.u.)	H(*r*) (a.u.)
Cs/Gr/di-hydrated Pb	0.053	0.162	– 0.008
Cs/Gr/di-hydrated Cu	0.049	0.258	0.001
Cs/GrO/di-hydrated Pb	0.071	0.205	– 0.015
Cs/GrO/di-hydrated Cu	0.117	0.581	– 0.306

### Calculated infrared frequencies

**Fig. 9:**
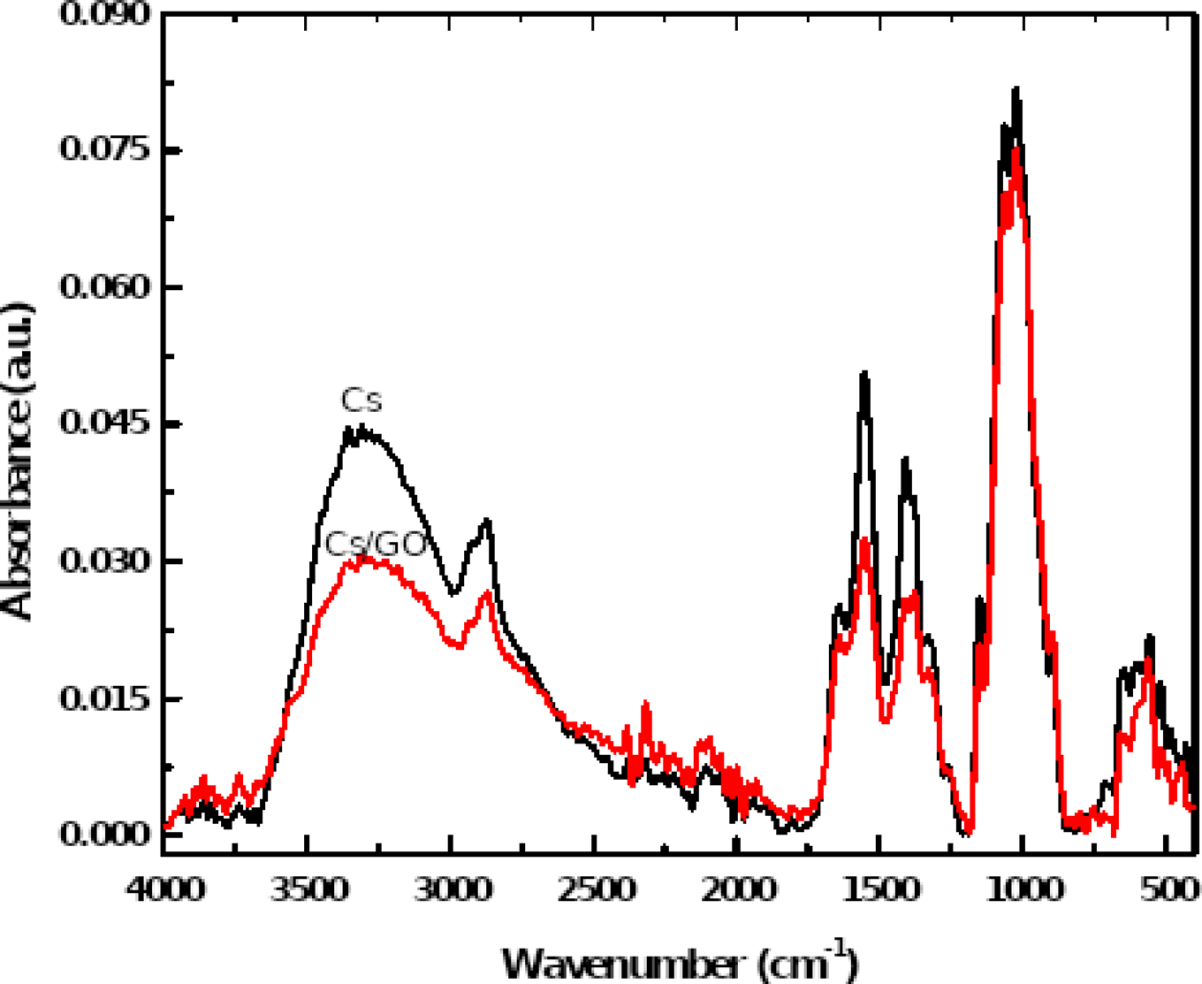
FTIR absorbance spectra of pure Cs and Cs/GrO composite.

Infrared frequencies of Cs/GrO were calculated using DFT: B3LYP/LANL2DZ basis set, then compared experimentally with those obtained with FTIR. Figure 9 display the absorbance spectra for Cs and Cs/GrO and Table 5 have the recorded frequencies and band assignments. Cs FTIR bands, the broad O-H stretching band and N-H stretching band were observed at the range 3355 ~ 3230 cm^-1^^61,62^. The band at 2925 ~ 2875 cm^-1^ is assigned to CH symmetric and asymmetric stretching^62,63^. The bands 1637 cm^-1^ and 1551 cm^-1^ corresponds to C = O stretching and N-H in amide I and amide II respectively^61,62^. Additionally, the CH_3_ band and C–O–C associate with 1387 cm^-1^ and 1022 cm^-1^ respectively. GrO FTIR bands such C–OH was observed at 3361 cm^-1^^64^, while C = C stretching observed at 1637 cm^-1^, 1799 cm^-1^ correspond to C = O stretching^65,66^. Finally, the bands at 1387 cm^-1^ and 1020 cm^-1^ can be assigned to C–H and O–C–O stretching as reported^64,65^. Table 6 compares the theoretical IR frequencies of Cs/GrO against the obtained FTIR. The results describe how closely the computed IR matching FTIR spectra that revealed the validity of the computational method.

**Table 5:** Band assignment for FTIR results of Cs and Cs/GrO.

Structure	FIIR	Assignment
Cs	3355 ~ 3230	N-H and O-H stretching
	2925 ~ 2875	CH symmetric and asymmetric stretching
	1637	C = O amide I
	1551	N–H amide II
	1387	CH_3_
	1022	C–O–C
GrO	3361	C–OH stretching
	1637	C = C stretching
	1799	C = O stretching
	1387	C–H deformation vibration
	1020	O–C–O stretching

**Table 6:** B3LYB/LANL2DZ calculated IR compared with FTIR for Cs/GrO.

LANL2DZ: IR	FTIR	Assignment
3725 ~ 3418	3355 ~ 3230	O–H stretching N–H stretching
3080	2925 ~ 2875	CH_2_ stretching
1670	1583	N–H amide II
1656 ~ 1581	1637	C = C stretching
1702	1799	C = O stretching

**Table 7:** Comparative performance of Cs/Gr and Cs/GrO for di-hydrated Pb and Cu.

Structure	E_a_ (eV)	TDM (Debye)	ΔE (eV)
Cs/Gr/di-hydrated Pb	– 13.869	9.220	0.675
Cs/Gr/di-hydrated Cu	– 13.689	8.111	2.613
Cs/GrO/di-hydrated Pb	– 12.975	13.309	0.919
Cs/GrO/di-hydrated Cu	– 14.211	3.629	1.937

## Conclusion

The reduction in ΔE and the increase in TDM indicate enhanced reactivity for the composites, combined with improvements in charge transfer as shown in the FMO and DOS analyses. Global reactivity descriptors complement ΔE and TDM results and suggest that the composites interacting with di-hydrated Pb exhibit higher reactivity. This highlights the superior electronic properties of Cs/Gr and Cs/GrO composites compared to their individual components. The adsorption energy (E_a_) values indicate exothermic interactions with both di-hydrated Pb and Cu, supporting the potential of these composites for heavy metal removal. A comparative summary of E_a_, TDM, and energy gap values for all systems is provided in Table 7, clearly illustrating the selective affinity of Cs/Gr for Pb and Cs/GrO for Cu. QTAIM analysis further confirmed these trends, revealing stable bonding interactions, with Cs/Gr forming stronger bonds with Pb and Cs/GrO with Cu. These findings align well with the adsorption energy results. The Cs/GrO composite was synthesized and characterized experimentally, and the FTIR analysis showed a close match with the calculated IR spectra, validating the computational approach.

## Data Availability

The data supporting the findings of this study can be obtained from the corresponding author upon request, subject to reasonable conditions.

## References

[1] Kharkan, J., Sayadi, M. H. & Rezaei, M. R. Investigation of heavy metals accumulation in the soil and pine trees. *Environ. Health Eng. Manag J.* **6**, 17–25 (2019). http://eprints.kmu.ac.ir/id/eprint/30840

[2] Gaur, N., Kukreja, A., Yadav, M. & Tiwari, A. Adsorptive removal of lead and arsenic from aqueous solution using Soya bean as a novel biosorbent: equilibrium isotherm and thermal stability studies. *Appl. Water Sci.* **8**, 98. 10.1007/s13201-018-0743-5 (2018).

[3] Ogunseitan, O. A. Public health and environmental benefits of adopting lead-free solders. *JOM* **59**, 12–17. 10.1007/s11837-007-0082-8 (2007).

[4] Wołowiec, M., Komorowska-Kaufman, M., Pruss, A., Rzepa, G. & Bajda, T. Removal of heavy metals and metalloids from water using drinking water treatment residuals as adsorbents: a review. *Minerals* **9**, 487 (2019). https://doi.org/10.3390/min9080487

[5] Malik, L. A., Bashir, A., Qureashi, A. & Pandith, A. H. Detection and removal of heavy metal ions: a review. *Environ. Chem. Lett.* **17**, 1495–1521. 10.1007/s10311-019-00891-z (2019).

[6] Taylor, A. A. et al. Critical review of exposure and effects: implications for setting regulatory health criteria for ingested copper. *Environ. Manag*. **65**, 131–159. 10.1007/s00267-019-01234-y (2020). 10.1007/s00267-019-01234-y PMC6960211 31832729

[7] Xu, X., Gao, B., Jin, B. & Yue, Q. Removal of anionic pollutants from liquids by biomass materials: a review. *J. Mol. Liq*. **215**, 565–595. 10.1016/j.molliq.2015.12.101 (2016).

[8] Aryee, A. A., Mpatani, F. M., Han, R., Shi, X. & Qu, L. A review on adsorbents for the remediation of wastewater: antibacterial and adsorption study. *J. Environ. Chem. Eng.* **9**, 109607. 10.1016/J.JECE.2021.106907 (2021).

[9] Neolaka, Y. A. B. et al. Potential of activated carbon from various sources as a low-cost adsorbent to remove heavy metals and synthetic dyes. *Results Chem.* **5**, 100711 10.1016/j.rechem.2022.100711 (2023).

[10] Bessa, R. A. et al. Hierarchical zeolite based on multiporous zeolite A and bacterial cellulose: an efficient adsorbent of Pb(II). *Microporous Mesoporous Mater.* **312**, 110752. 10.1016/j.micromeso.2020.110752 (2021).

[11] Mehrabi, N., Haq, A., Aich, N. & U. F. & Application of deep eutectic solvent for conjugation of magnetic nanoparticles onto graphene oxide for lead(II) and methylene blue removal. *J. Environ. Chem. Eng.* **25**, 104222. 10.1016/j.jece.2020.104222 (2020).

[12] Liang, S. et al. Polyester nanofilms with enhanced polyhydroxyl architectures for the separation of metal ions from aqueous solutions. *ACS Appl. Nano Mater.* **1**, 6176–6186. 10.1021/acsanm.8b01325 (2018).

[13] Nadeem, M. et al. Sorption of lead from aqueous solution by chemically modified carbon adsorbents. *J. Hazard. Mater.* **138**, 604–613. 10.1016/j.jhazmat.2006.05.098 (2006). 16839677 10.1016/j.jhazmat.2006.05.098

[14] Dehabadi, L. & Wilson, L. D. Polysaccharide-based materials and their adsorption properties in aqueous solution. *Carbohydr. Polym.* **113**, 471–479. 10.1016/j.carbpol.2014.06.083 (2014). 25256509 10.1016/j.carbpol.2014.06.083

[15] Alhwaige, A. A. Novel biobased chitosan/polybenzoxazine cross-linked polymers and advanced carbon aerogels for CO₂ adsorption. Ph.D. Thesis, Case Western Reserve University, Cleveland, OH, USA (2014). https://etd.ohiolink.edu/pg_10?0::NO:10:P10_ACCESSION_NUM:case1396437860

[16] Tiwari, S. K. et al. Magical allotropes of carbon: prospects and applications. *Crit. Rev. Solid State Mater. Sci.* **41**, 257–317. 10.1080/10408436.2015.1127206 (2016).

[17] Obodo, R. M., Ahmad, I. & Ezema, F. I. Introductory chapter: graphene and its applications. In: (ed Ezema, F. I.) Graphene and its Derivatives—Synthesis and Applications, 1–9 (IntechOpen, 2019).

[18] Siwal, S. S., Zhang, Q., Devi, N. & Thakur, K. V. Carbon-based polymer nanocomposite for high-performance energy storage applications. *Polymers* **12**, 30505. 10.3390/polym12030505 (2020). 10.3390/polym12030505 PMC7182882 32110927

[19] Liu, X. et al. Graphene oxide-based materials for efficient removal of heavy metal ions from aqueous solution: a review. *Environ. Pollut*. **252**, 62–73 (2019). 31146239 10.1016/j.envpol.2019.05.050

[20] Ghulam, A. N. et al. Graphene oxide (GO) materials—applications and toxicity on living organisms and environment. *J. Funct. Biomater.* **13**, 77. 10.3390/jfb13020077 (2022). 35735932 10.3390/jfb13020077 PMC9224660

[21] Yan, Y. et al. Synthesis of graphene: potential carbon precursors and approaches. *Nanotechnol Rev.* **9**, 1284–1314. 10.1515/ntrev-2020-0100 (2020).

[22] Asghar, F. et al. Fabrication and prospective applications of graphene oxide-modified nanocomposites for wastewater remediation. *RSC Adv.* **12**, 11750–11768. 10.1039/D2RA00271J (2022). 35481102 10.1039/d2ra00271j PMC9016740

[23] Gao, W. The chemistry of graphene oxide. In: Graphene Oxide: Reduction Recipes, Spectroscopy, and Applications. 61–95 (Springer, 2015). 10.1007/978-3-319-15500-5

[24] Ramesha, G. K., Kumara, V., Muralidhara, A. V., Sampath, S. & H. B. & Graphene and graphene oxide as effective adsorbents toward anionic and cationic dyes. *J. Colloid Interface Sci.* **361**, 270–277 (2011). 21679961 10.1016/j.jcis.2011.05.050

[25] Xu, L. & Wang, J. The application of graphene-based materials for the removal of heavy metals and radionuclides from water and wastewater. *Crit. Rev. Environ. Sci. Technol.* **47**, 1042–1105 (2017).

[26] Shinya, S. & Fukamizo, T. Interaction between Chitosan and its related enzymes: a review. *Int. J. Biol. Macromol.*10.1016/j.ijbiomac.2017.02.040 (2017). 28223213 10.1016/j.ijbiomac.2017.02.040

[27] Duri, S. & Tran, C. D. Supramolecular composite materials from cellulose, chitosan, and cyclodextrin: facile preparation and their selective inclusion complex formation with endocrine disruptors. *Langmuir* **29**, 5037–5049.10.1021/la3050016 (2013). 23517477 10.1021/la3050016 PMC3640277

[28] Kumari, A. R. & Sobha, K. Removal of lead by adsorption with the renewable biopolymer composite of feather (Dromaius novaehollandiae) and Chitosan (Agaricus bisporus). *Environ. Technol. Innov.* **6**, 11–26. 10.1016/j.eti.2016.04.004 (2016).

[29] Salehi, E., Daraei, P. & Shamsabadi, A. A. A review on chitosan-based adsorptive membranes. *Carbohydr. Polym.* **152**, 419–432. 10.1016/j.carbpol.2016.07.033 (2016). 27516289 10.1016/j.carbpol.2016.07.033

[30] Alhwaige, A. A., Agag, T., Ishida, H. & Qutubuddin, S. Biobased chitosan/polybenzoxazine cross-linked films: preparation in aqueous media and synergistic improvements in thermal and mechanical properties. *Biomacromolecules* **14**, 1806–1815. 10.1021/bm4002014 (2013). 23631553 10.1021/bm4002014

[31] Wu, F. C., Tseng, R. L. & Juang, R. S. Kinetic modeling of liquid-phase adsorption of reactive dyes and metal ions on Chitosan. *Water Res.* **35**, 613–618 (2001). 11228956 10.1016/s0043-1354(00)00307-9

[32] Ren, H. et al. Efficient lead ion removal from water by a novel Chitosan gel-based sorbent modified with glutamic acid ionic liquid. *Carbohydr. Polym.* **207**, 737–746. 10.1016/j.carbpol.2018.12.043 (2019). 30600060 10.1016/j.carbpol.2018.12.043

[33] Rebello, S. et al. Chitosan a versatile adsorbent in environmental remediation in the era of circular economy—a mini review. *Sustain. Chem. Pharm.* **32**, 101004. 10.1016/j.scp.2023.101004 (2023).

[34] Wang, Z., Wang, Y. & Yao, C. Research progress in the treatment of uranium(VI)-contaminated wastewater by modified Chitosan. *J. Radioanal Nucl. Chem.*10.1007/s10967-021-08010-5 (2021).

[35] Hawick, K., Grove, D., Coddington, P. & Buntine, M. Commodity cluster computing for computational chemistry. *Internet J. Chem.* **3**, 1099–8292 (2000).

[36] Omar, A. et al. Investigation of morphological, structural and electronic transformation of PVDF and zno/rgo/pvdf hybrid membranes. *Opt. Quantum Electron.* **55**, 381. 10.1007/s11082-023-04663-6 (2023). DOI:

[37] El-Mansy, M. A., Bayoumy, A. M., Elhaes, H. & Ibrahim, M. A. Exploring the electronic, optical, and bioactive properties for new modified fullerenes via molecular modeling. *Opt. Quantum Electron.* **55**, 100. 10.21203/rs.3.rs-218502/v1 (2023).

[38] Brandenburg, J. G. & Grimme, S. Dispersion corrected Hartree–Fock and density functional theory for organic crystal structure prediction. In: *Prediction and calculation of crystal structures*. *Top. Curr. Chem.* **345**, 1–23. 10.1007/128_2013_488 (2014). 24220994 10.1007/128_2013_488

[39] Taniş, E. A. A study of silicon and germanium-based molecules in terms of solar cell devices performance. *Turk. J. Chem.* **46**, 1607–1619 (2022). https://doi.org/10.55730/1300-0527.3464 37529753 10.55730/1300-0527.3464 PMC10390171

[40] Frisch, M. J. et al. Gaussian 09, Revision C.01, Gaussian, Inc., Wallingford, CT, USA (2010).

[41] Petersson, G. A. & Al-Laham, M. A. A complete basis set model chemistry. II. Open-shell systems and the total energies of the first-row atoms. *J. Chem. Phys.* **94**, 6081–6090. 10.1063/1.460447 (1991).

[42] Becke, A. D. Density-functional thermochemistry. I. The effect of the exchange-only gradient correction. *J. Chem. Phys.* **96**, 2155–2160. 10.1063/1.462066 (1992).

[43] Lee, C., Yang, W. & Parr, R. G. Development of the Colle-Salvetti correlation-energy formula into a functional of the electron density. *Phys. Rev. B*. **37**, 785–789. 10.1103/PhysRevB.37.785 (1988). 10.1103/physrevb.37.785 9944570

[44] Lassoued, M. S. et al. Synthesis, crystal structure, DFT (B3LYP/LanL2DZ) and photoluminescence study of new stanate (IV) based inorganic-organic hybrid. *J. Phys. Chem. Solids*. **121**, 177–185. 10.1016/j.jpcs.2018.05.024 (2018).

[45] Check, C. E. et al. Addition of polarization and diffuse functions to the LANL2DZ basis set for P-Block elements. *J. Phys. Chem. A*. **105**, 8111–8116. 10.1021/jp011945l (2001).

[46] Eryılmaz, S. The theoretical investigation of global reactivity descriptors, NLO behaviours and bioactivity scores of some norbornadiene derivatives. *Sakarya Univ. J. Sci.* **22**, 1638–1647. 10.16984/saufenbilder.359837 (2018).

[47] Obot, I. B., Macdonald, D. D. & Gasem, Z. M. Density functional theory (DFT) as a powerful tool for designing new organic corrosion inhibitors. Part 1: an overview. *Corros. Sci.* **99**, 1–30. 10.1016/j.corsci.2015.01.037 (2015).

[48] Lu, T. & Chen, F. Multiwfn: a multifunctional wavefunction analyzer. *J. Comput. Chem.* **33**, 580–592. 10.1002/jcc.22885 (2012). 22162017 10.1002/jcc.22885

[49] Humphrey, W., Dalke, A. & Schulten, K. V. M. D. Visual molecular dynamics. *J. Mol. Graph*. **14**, 33–38. 10.1016/0263-7855(96)00018-5 (1996). 8744570 10.1016/0263-7855(96)00018-5

[50] Amin, K. S. et al. Design and implementation of pla/go/metal oxide composites for CO₂ sensing application. *Sci. Rep.* **15**, 1–12. 10.1038/s41598-025-89337-x (2025). 39962159 10.1038/s41598-025-89337-x PMC11832736

[51] Ezzat, H. A. et al. Molecular modeling analyses of functionalized cellulose. *Sci. Rep.* **14**, 27698. 10.1038/s41598-024-77629-7 (2024). 39532973 10.1038/s41598-024-77629-7 PMC11557594

[52] Sabry, N. M. et al. Electronic structure, global reactivity descriptors and nonlinear optical properties of Glycine interacted with zno, MgO and CaO for bacterial detection. *Sci. Rep.* **14**, 22801. 10.1038/s41598-024-72846-6 (2024). 39353963 10.1038/s41598-024-72846-6 PMC11445471

[53] Ibrahim, M. A. et al. On the spectroscopic analyses of [α(2,5 dimethylfuryl) ethylidene] (dicyclopropyl methylene) 2,5 Furadione. *Spectrochim Acta Part. A*. **1**, 1. 10.1016/j.saa.2011.11.039 (2012). 10.1016/j.saa.2011.11.039 22154265

[54] Politzer, P., Laurence, P. R. & Jayasuriya, K. Molecular electrostatic potentials: an effective tool for the Elucidation of biochemical phenomena the meaning and use of the electrostatic potential definition and significance. *Environ. Health Perspect.* **61**, 191. 10.1289/ehp.8561191 (1985). 2866089 10.1289/ehp.8561191 PMC1568763

[55] Cheung, D. L. & Troisi, A. Theoretical study of the organic photovoltaic electron acceptor PCBM: morphology, electronic structure, and charge localization. *J. Phys. Chem. C*. **114**, 20479–20488. 10.1021/jp1049167 (2010).

[56] Hassan, B. et al. A DFT based analysis of adsorption of hg²⁺ ion on Chitosan monomer and its citralidene and Salicylidene derivatives: prior to the removal of Hg toxicity. *Int. J. Biol. Macromol.* **99**, 549–554. 10.1016/j.ijbiomac.2017.03.032 (2017). 28283449 10.1016/j.ijbiomac.2017.03.032

[57] Shahzad, A. et al. Heavy metals removal by EDTA-functionalized Chitosan graphene oxide nanocomposites. *RSC Adv.* **7**, 9764–9771. 10.1039/C6RA28406J (2017).

[58] Li, L., Zhao, L., Ma, J. & Tian, Y. Preparation of graphene oxide/chitosan complex and its adsorption properties for heavy metal ions. *Green. Process. Synth.* **9**, 294–303. 10.1515/gps-2020-0030 (2020).

[59] Bader, R. F. W. A quantum theory of molecular structure and its applications. *Chem. Rev.* **91** (5), 893–928. 10.1021/cr00005a013 (1991).

[60] Adalikwu, S. A. et al. B- and Al-Doped porous 2D covalent organic frameworks as nanocarriers for Biguanides and Metformin drugs. *ACS Appl. Bio Mater.* **5**, 5887–5900. 10.1021/acsabm.2c00855 (2022). 36413624 10.1021/acsabm.2c00855

[61] Elhaes, H. et al. Hartree–Fock and DFT study of the molecular structure and electronic properties of functionalized Chitosan and Chitosan-graphene oxide for electronic applications. *Opt. Quantum Electron.* **56**10.1007/s11082-023-05978-0 (2024).

[62] Lustriane, C. et al. Effect of Chitosan and Chitosan-nanoparticles on post harvest quality of banana fruits. *J. Plant. Biotechnol.* **45**, 36–44. 10.5010/jpb.2018.45.1.036 (2018).

[63] Reicha, F. M., Sarhan, A., Abdel-Hamid, M. I. & El-Sherbiny, I. M. Preparation of silver nanoparticles in the presence of Chitosan by electrochemical method. *Carbohydr. Polym.* **89**, 236–244. 10.1016/j.carbpol.2012.03.002 (2012). 24750629 10.1016/j.carbpol.2012.03.002

[64] Faniyi, I. O. et al. The comparative analyses of reduced graphene oxide (RGO) prepared via green, mild and chemical approaches. *SN Appl. Sci.* **1**10.1007/s42452-019-1188-7 (2019).

[65] Kanta, U. et al. Preparations, characterizations, and a comparative study on photovoltaic performance of two different types of Graphene/TiO₂ nanocomposites photoelectrodes. *J. Nanomater.* 1–13 (2017). (2017). 10.1155/2017/2758294

[66] Esmaeili, Y. et al. Graphene oxide and its derivatives as promising in-vitro bio-imaging platforms. *Sci. Rep.* **10**10.1038/s41598-020-75090-w (2020). 10.1038/s41598-020-75090-w PMC7582845 33093483

